# Pt-N Co-Modified TiO_2_ Nanotube Electrode Photoelectrocatalytic Degradation of Oxytetracycline in Simulated Wastewater

**DOI:** 10.3390/toxics10110635

**Published:** 2022-10-22

**Authors:** Liming Wang, Mengyao Li, Liang Pei, Tingting Liu, Tian Zhang, Dong Ao

**Affiliations:** 1 College of Environmental and Chemical Engineering, Xi’an Polytechnic University, Xi’an 710048, China; 2 Xinjiang Institute of Ecology and Geography, Chinese Academy of Sciences, Urumqi 830011, China; 3 Institute of Geographic Sciences and Natural Resources Research, Chinese Academy of Sciences, Beijing 100101, China

**Keywords:** photoelectrocatalysis, Oxytetracycline, Pt-N co-modified TiO_2_ nanotubes, photodeposition

## Abstract

Using photodeposition and plasma, Pt-N co-modified TiO_2_ nanotube electrodes were created. Several techniques, such as SEM, XRD, UV-VIS-DRS, XPS, and PL, were used to analyze the electrode shape, crystalline structure, light absorption range, elemental composition, and photogenerated carrier recombination efficiency. Using the electrochemical workstation, EIS and I-t were utilized to examine the electrochemical characteristics. The results indicated that the diameter of the TiO_2_ nanotube tubes was around 90 nm, and that the photodeposition duration affected the amount of Pt particles deposited. The deposited Pt particles efficiently reduced the photogenerated carrier complexation rate of the N-TiO_2_ nanotube electrode, contributing to the separation of electron-hole pairs and light utilization. Electrochemical studies indicated that Pt-N co-modified TiO_2_ increased the electrode’s oxidation and electrical conductivity, as well as its photoelectrocatalytic capacity. Oxytetracycline degradation in simulated wastewater by a Pt-N co-modified TiO_2_ nanotube electrode revealed the exceptional PEC activity, and the oxytetracycline degradation processes followed primary kinetics. •O_2_^−^ and •OH played a significant role in the photoelectrocatalytic degradation of oxytetracycline, resulting in a novel method for oxytetracycline degradation.

## 1. Introduction

Antibiotics are emerging organic pollutants that are gaining widespread attention and importance from environmentalists [1]. Oxytetracycline (OTC), a popular tetracycline antibiotic, is used extensively in the pharmaceutical and agricultural industries. However, the majority of OTC is not completely absorbed and utilized and enters environmental units such as surface water, groundwater, and soil through sewage discharge, surface runoff, and waste leachate in livestock [2,3,4]. OTC has currently been found in a number of environmental media, and as it continues to build up there, it creates bacteria that are resistant to it, which threatens the balance of microbial ecosystems and, in turn, indirectly jeopardizes human safety [5,6]. Conventional wastewater treatment methods are challenging to completely eliminate, and antibiotic wastewater limits the growth of, bacteria in wastewater treatment, leading to considerable changes in COD/BOD concentrations and unstable water quality [7,8]. Assume that during the wastewater’s pretreatment stage, the antibiotics are broken down and lessen the growth inhibition on the microbes. The antibiotics must be eliminated before discharge into the environment [9,10]. 

Due to high catalytic efficiency and environmental friendliness, semiconductor photocatalysis has attracted a lot of interest in recent years for cleaning organic wastewater [11]. Applying bias pressure to a photocatalytic system allows for the separation of photo-generated electrons and hole pairs, which contributes to the production of free radicals. This method combines photochemistry and electrochemistry to increase the degradation efficiency of pollutants. TiO_2_ nanotube arrays in situ prepared on titanium sheets are of interest due to their chemical stability, resistance to photocorrosion, high charge transport, non-toxicity, and low cost, compared to other nanotube electrodes loaded with semiconductor materials [12]. They also have several advantages, including the possibility to use the titanium substrate as an electrode in addition to the surface-generated TiO_2_. However, TiO_2_ nanoparticles’ low optical quantum efficiency for visible light has restricted their general applicability as optoelectronic materials [13]. Numerous techniques, including metal particle modification [14,15], nonmetallic ion modification [16,17], semiconductor compounding [18,19], etc., have been used to increase TiO_2_’s effective utilization of visible light. These techniques aim to increase the material’s absorption of visible light, broaden its absorption spectrum, and increase its optical quantum efficiency, as well as to enhance the separation of photogenerated electron-hole carriers and lengthen the carrier lifespan [13]. 

TiO_2_ photocatalysts have also been successfully modified using single approaches, although they all have flaws. To achieve co-doping modification of TiO_2_, a variety of single approaches can be combined, making up for each other’s shortcomings and boosting photocatalytic activity. To improve the single-doping photocatalytic efficiency, Tio [20], for instance, prepared Fe-N co-doped TiO_2_ photocatalysts using the sol–gel method and showed good degradation performance in the degradation of Acid Orange 7 azo dye. Nzaba and co-workers [21] used a template-assisted method to prepare Pt, N co-doped TiO_2_ for degradation with photo-bright black. Ghoreishian [22] used electrostatic spinning to prepare Fe-Sn co-doped TiO_2_ nanofibers to simulate visible-light degradation of tetracycline. 

N and O have more similar atomic radii and can penetrate the TiO_2_ lattice more quickly than other ions; therefore, N doping efficiency is better than other ion doping, according to several ion doping studies [16,23,24]. The stability and surface plasmon resonance effects of noble metals are advantageous for separating electron-hole pairs and extending the range of light absorption [25]. Due to their greater catalytic activity and superior capacity to grab electrons, precious metals placed on TiO_2_ materials can efficiently separate photogenerated electron-hole pairs. The strongest Schottky barrier is created between Pt particles and TiO_2_, prohibiting the complexation of electron-hole pairs and electron, which has been claimed to make Pt one of the most active metals for photocatalytic enhancement [26]. 

Based on the previous group’s inquiry into the ideal N-modified TiO_2_ nanotubes, Pt-N co-modified TiO_2_ nanotube electrodes were prepared in this study. N doping can effectively reduce the forbidden band width of TiO_2_, thus improving the absorption intensity of the TiO_2_ catalyst in the visible-light region; Pt deposition can not only capture the electrons in the conduction band, so that fewer photogenerated electrons can compound with holes, but also can generate a large number of electrons to the conduction band under visible-light excitation by the plasmon resonance effect and participate in the degradation reaction of organic matter. The aim is to improve the utilization of sunlight and the quantum yield of the catalyst, so as to improve the degradation effect of the catalyst on OTC, and to provide an experimental and theoretical basis for photoelectrocatalysis oxidation for the removal of hard-to-degrade antibiotics in water.

## 2. Materials and Methods

### 2.1. Reagents

Oxytetracycline (C_2_H_24_N_2_O_9_) was purchased from Sinopharm Chemical Reagents Co., Ltd. (Shanghai, China); Chloro-platinic acid (H_2_PtCl_6_·6H_2_O) was purchased from Sinopharm Chemical Reagents Co., Ltd.; Acetone (C_2_H_6_O) was purchased from Tianjin Li’an Longhua Pharmaceutical Chemical Co., Ltd. (Tianjin, China); Hydrofluoric acid (HF) was purchased from the Xi’an Chemical Reagent Factory (Xi’an, China); Nitric acid (HNO_3_) was purchased from the Tianjin Damao Chemical Reagent Factory (Tianjin, China); Anhydrous ethanol (C_2_H_6_O) was purchased from Tianjin Fuyu Fine Chemical Co., Ltd. (Tianjin, China); Ammonium fluoride (NH_4_F) was purchased from the Xi’an Chemical Reagent Factory. Phosphoric acid (H_3_PO_4_) was purchased from the Xi’an San Pu Fine Chemical Plant (Xi’an, China); Isopropanol (C_3_H_8_O) was purchased from Tianjin Comio Chemical Reagent Co., Ltd. (Tianjin, China); Ammonium oxalate ((NH_4_)_2_C_2_O_4_) was purchased from Tianjin Kemiou Chemical Reagent Co., Ltd. (Tianjin, China); P-benzoquinone (C_6_H_4_O_2_) was purchased from Shanghai Titan Technology Co., Ltd. (Shanghai, China). All chemicals involved in our work were of ana-lytical reagent grade and applied without any further purification processes.

### 2.2. Preparation of Pt-N-TiO_2_ Nanotube Electrode

The Ti flakes were pretreated using anhydrous ethanol, acetone, and pure water in an ultrasonic cleaner for 1 h. The surface oxidation layer was removed from the Ti flakes using an acid washing solution (HF: HNO_3_: H_2_O = 1:4:5), and TiO_2_ nanotubes were prepared by anodic oxidation in a fluorine-containing electrolyte for 20 min (voltage 20 V). The anodized TiO_2_ nanotubes were immersed in a chloroplatinic acid solution (1 g/L) and deposited in the photoreactor at different times (power 500 W). The residual solution of the electrode was cleaned using anhydrous ethanol, dried, and processed for use. The spare electrode was treated in a low-temperature plasma discharge apparatus filled with N_2_ atmosphere (time 90 s, power 20 w, air pressure 20 pa), and the finished electrode was treated in a muffle furnace at a high temperature for 2 ha (temperature 500 °C, heating rate 5 °C/min).

### 2.3. Photoelectrocatalytic Degradation of Oxytetracycline

The photoelectrocatalytic OTC experiment was performed in a homemade reaction chamber by adding 250 mL of OTC solution, applying a specific voltage (25 V) between two electrodes (the cathode was the platinum electrode and the anode was the working electrode), and turning on a long-arc mercury lamp. Electrocatalytic degradation (EC) was performed under the condition of maintaining voltage but turning off the light source; photocatalytic degradation (PC) was performed under the condition of turning on the light source without adding voltage. Samples were taken every ten minutes, and absorbance tests were performed to determine the degradation rate. The degradation rate (η) and the kinetic constant of the primary reaction (k) of OTC were calculated using Equations (1) and (2), respectively

H = (C_0_ − C_t_)/C_0_ × 100% (1)

In(C_0_/C_t_) = kt (2)

where C_0_ and C_t_ are the initial concentration of OTC (mg/L) and the concentration at photoelectrocatalysis degradation time t(min), respectively, and k is the primary kinetic reaction rate constant (min^−1^).

### 2.4. Characterization Tests

The morphology of TiO_2_ nanotubes was examined using field emission scanning electron microscopy (SEM) both before and after modification. The crystalline modifications in nanotubes both before and after conversion were characterized using X-ray diffraction (XRD), and the elemental species of nanotube electrodes were described using X-ray photoelectron spectroscopy (XPS). The light absorption degree of the electrode was characterized by UV-VIS diffuse reflectance spectrum (UV-VIS-DRS). Prior to and following alteration, their photogenerated carrier complex rates were compared using photoluminescence spectroscopy (PL). Three electrodes were employed in a three-electrode system, and electrochemical workstation tests were used to analyze the electrodes’ electrochemical characteristics (electrolyte concentration was 0.5 mol/L NaSO_4_). Testing OTC absorbance was conducted with the MAPADA P5 UV-VIS spectrophotometer.

## 3. Results and Discussion

### 3.1. Scanning Electron Microscopy

**Figure 1:**
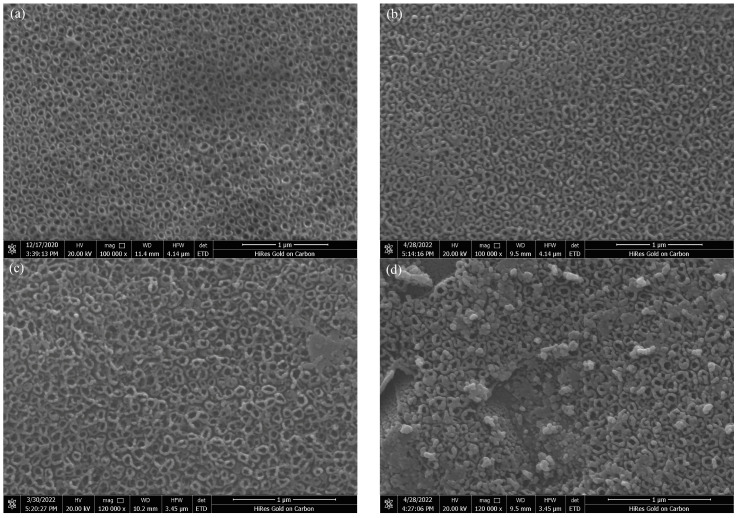
SEM patterns of Pt-N-TiO_2_ nanotube electrodes with different deposition times: (**a**) TiO_2_; (**b**) 30 min Pt-N-TiO_2_; (**c**) 60 min Pt-N-TiO_2_; (**d**) 90 min Pt-N-TiO_2_.

SEM analysis was used to compare the microstructure of the Pt-N co-modified nanotube electrode before and after synthesis. The nanotube electrode before modification is shown in Figure 1a. The arrangement structure is tight and orderly, the nanotube morphology is apparent, the tube diameter is around 90 nm, and the Pt-N co-modification did not alter the nanotube morphology. The Pt-N co-modified TiO_2_ nanotube electrode is depicted in Figure 1b–d with various photodeposition times. The Figure shows the effect of deposition time on the amount of deposited Pt particles. The longer the deposition time, the more Pt nanoparticles are deposited, and it is simple for them to aggregate and block the nanotube orifice, which could have an impact on the photoelectrocatalysis performance.

### 3.2. X-ray Photoelectron Spectroscopy

**Figure 2:**
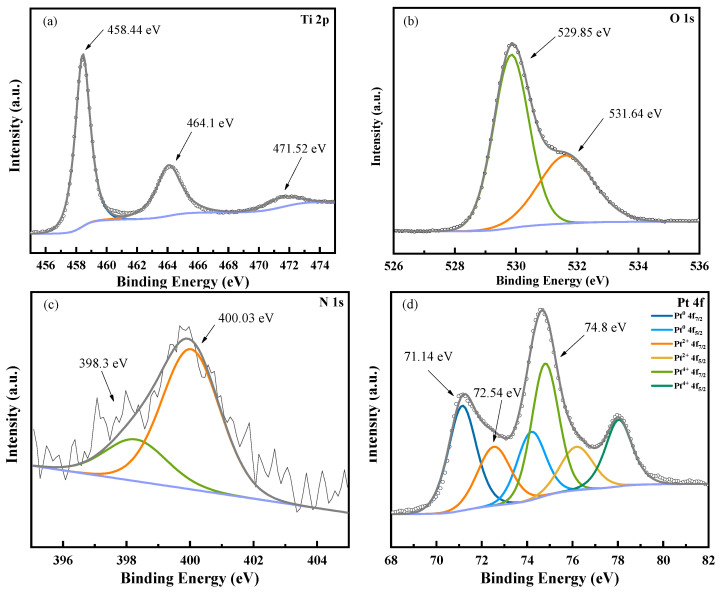
XPS pattern of Pt-N-TiO_2_ nanotube electrode: (**a**) Ti 2p; (**b**) O 1s; (**c**) N 1s; (**d**) Pt 4f.

Pt, N, Ti, and O are present, according to XPS analysis. The Ti 2p, O 1s, Pt 4f and N 1s spectral areas were subjected to numerous high-resolution scans in order to evaluate the respective elemental composition ratios. Ti accounted for 13.2%, O accounted for 43.81%, C accounted for 37.05%, N accounted for 2.1%, Pt accounted for 3.84%. Element C may have adsorbed C compounds from the air during the sitting of the sample. The predominant elements are Ti and O. The O 1s 529.85 eV is mainly attributed to TiO_2_ formation and the regional spectrum, and N 1s at characteristic peaks of 398.03 eV and 400.03 eV, where 398.07 eV corresponds to the valence bonds in the form of substituted N-Ti-N and Ti-N-O, suggesting that the N in the co-doping is replaced by the presence of lattice oxygen in TiO_2_ [27]. Figure 2d shows the XPS pattern of the Pt element with binding energies of 71.14 eV, 72.54 eV, and 74.8 eV for Pt 4f_7/2_ photoelectron peaks, which indicates that the Pt element valence is gradually reduced from the Pt^4+^ to the Pt^0+^ state and is present at the mouth of the TiO_2_ nanotubes in the form of singlet Pt, which is consistent with the SEM characterization results.

### 3.3. X-ray Diffraction and UV-VIS Diffuse Reflectance Specatrum

**Figure 3:**
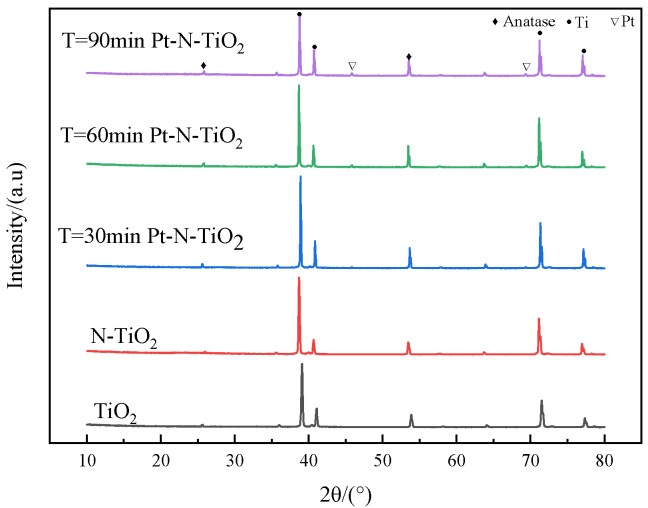
XRD pattern of each nanotube.

As seen in Figure 3, all of the peaks in the XRD patterns of Pt-N co-modified nanotube electrodes with various photodeposition times show good indications. There are apparent diffraction peaks of Ti in the figure, 2θ = 25.37°and 54.02° are the characteristic diffraction peaks of TiO_2_ anatase 101 crystal plane as well as 105 crystal plane (PDF 02-0406). The characteristic diffraction peaks of noble metal Pt appear at 2θ = 46.2°and 67.5°. It is interesting to note that the intensity of the separate peaks also increases with the increasing of deposition time, suggesting that more Pt monomers are reduced. This is consistent with the literature’s description [28].

**Figure 4:**
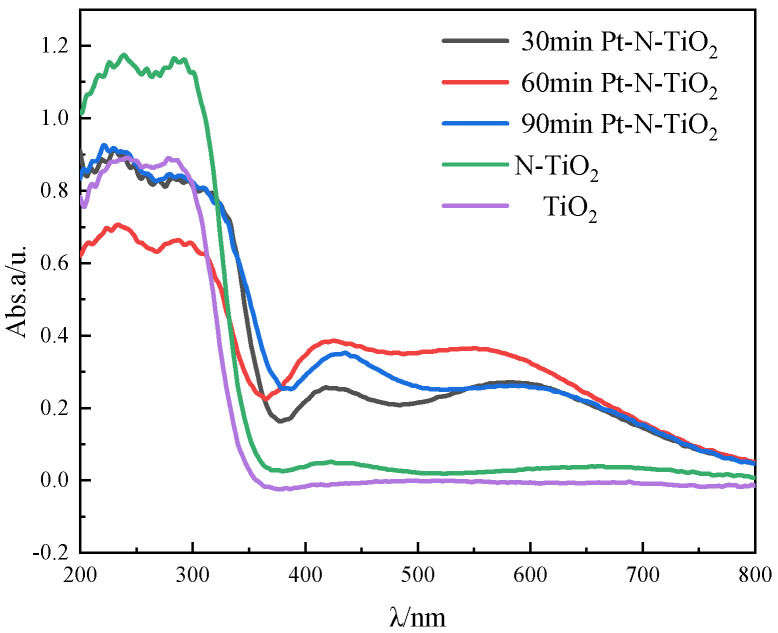
UV−VIS−DRS pattern of each nanotube.

According to the figure, which displays the UV-VIS-DRS patterns of Pt-N co-modified nanotube electrodes with various deposition rates, it is essential to analyze the light absorption performance of nanotube electrodes when studying photocatalytic performance. A wavelength range of 200–800 nm was selected for the diffuse reflection performance test of the samples. In Figure 4, the UV-VIS-DRS profiles of nanotube electrodes with various deposition times are displayed. Comparing the modified nanotube electrode to the TiO_2_ nanotube electrode, it is clear that the modified nanotube electrode absorbs visible light more effectively. The Pt nanoparticles put on the surface of the nanotube film led to red-shifted. The plasma resonance effect (SPR), which happens when the extinction wave and plasma wave resonate across a medium with sparse light, causes photons to rush to the surface plasma and weaken the light that is reflected, enhancing light absorption as a result [29].

### 3.4. Photoluminescence Spectroscopy

**Figure 5:**
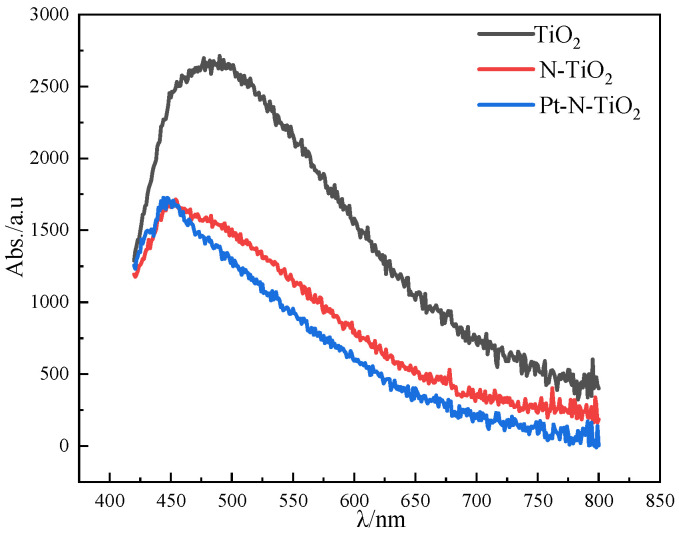
PL pattern of each nanotube.

By measuring the lifetimes of electron-hole pairs using PL spectra, it was possible to learn more about how TiO_2_, N-TiO_2_, and Pt-N-TiO_2_ nanotube electrodes affected the development of photoexcited carriers and the composite dynamics. The PL spectra of each nanotube electrode are shown in Figure 5, and an excitation pulse at a wavelength of 420 nm was used to test several TiO_2_ nanotubes. The experimental results show that the fluorescence spectra of TiO_2_ nanotubes, N-TiO_2_ nanotubes, and Pt-N-TiO_2_ nanotubes decreased in the order of intensity for the three materials, indicating that the recombination of electron and hole pairs is reduced of the modified TiO_2_ nanotube electrodes; in addition, the Pt -N-TiO_2_ nanoscopic electrode is much less intense than the pure TiO_2_ nanotube electrode, and when Pt is in contact with TiO_2_, a Schottky barrier is formed. TiO_2_ serves as a photoexcited electron source, and Pt serves as an electron trap. High electron-affinity Pt nanoparticles can reduce the recombination of electron and hole pairs, increasing photoactivity [30]. They should show the best photoelectrocatalysis performance since they have the longest electron and hole-pairs lifetime.

### 3.5. Electrochemical Analyses

**Figure 6:**
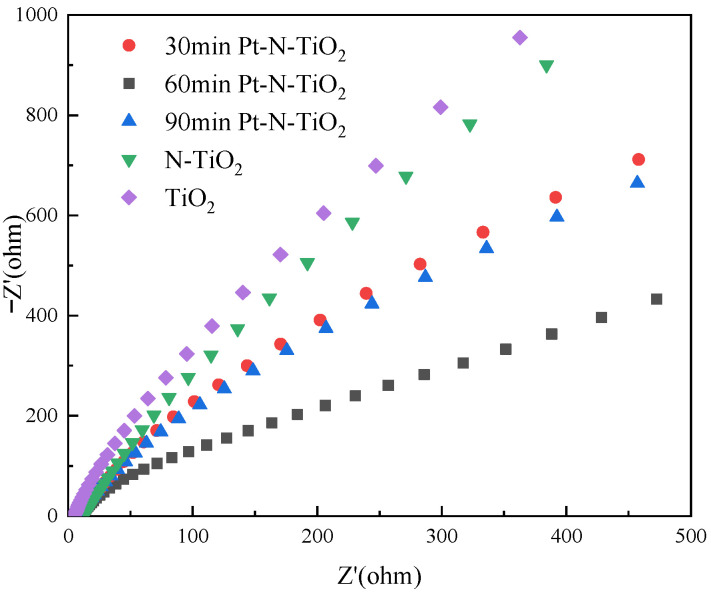
EIS pattern of each nanotube.

To study the charge transfer characteristics and efficiency of the electrolyte-electrode interface, electrochemical impedance spectroscopy (EIS) tests were carried out to produce Nyquist plots. The results are displayed in Figure 6. Each sample typically has a near-semicircular orbital; the semicircular radius is related to the charge transfer resistance; the electrode with the smaller arc radius, on the other hand, has a faster interfacial electron transfer and a longer induced electron-hole pairs lifetime. The energy barrier to be overcome is also lower, achieving effective charge separation and enabling better electron transport properties for this electrode material [31]. The Pt-N co-modified TiO_2_ nanotube electrodes all exhibit a smaller half-arc diameter, which indicates that the surface-modified Pt nanoparticles effectively promote carrier separation and interfacial charge transfer efficiency, with the nanotube electrode with a photodeposition Pt time of 60 min having the slightest slope, indicating the best photoelectrocatalysis activity.

**Figure 7:**
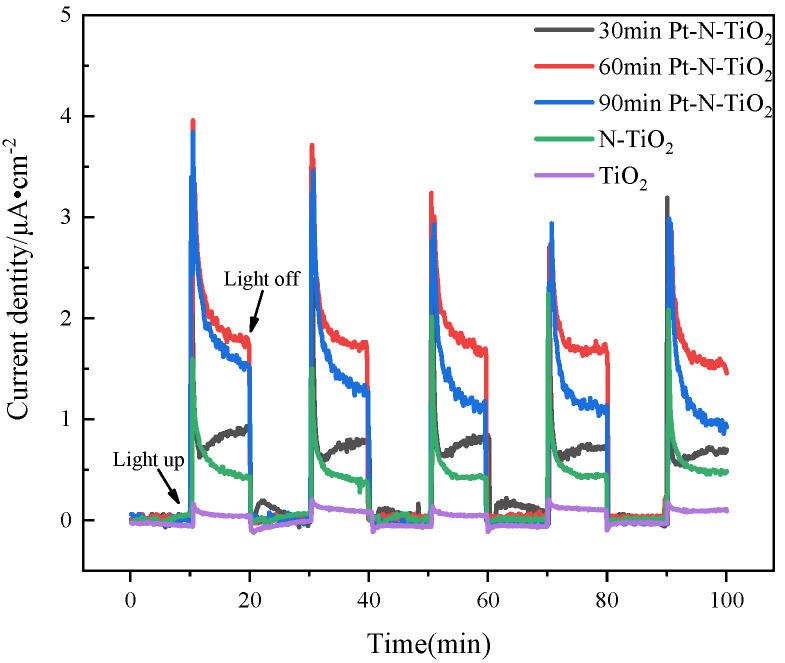
I-t pattern of each nanotube.

The photocurrent distribution is displayed in Figure 7 to help understand the impact of Pt-N loading on the interfacial electron transfer of the TiO_2_ solution. In comparison to N-TiO_2_ and TiO_2_, the photocurrent response values of Pt-N co-modified TiO_2_ nanotube electrodes are typically between four and six times greater. The maximum photocurrent response after 60 min of deposition shows that the right quantity of Pt deposition facilitates and can even boost photocurrent by making the phototransfer of electrons very simple. By trapping electrons to postpone the formation of the electron-hole pairs recombination, Pt nanoparticles speed the interfacial electron transfer [32].

### 3.6. Photoelectrocatalytic Degradation of Oxytetracycline

**Figure 8:**
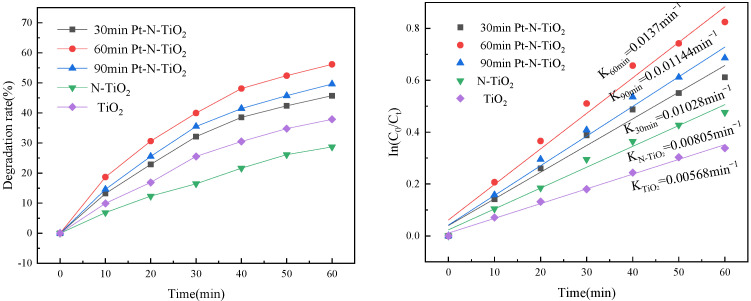
Degradation curves and reaction kinetics of photoelectrocatalysis OTC with different TiO_2_ nanotube electrodes.

OTC (30 mg/L; pH = 10; Time:60 min) was used as target pollutants to evaluate the photoelectrocatalytic activity. The photocatalytic OTC curves of Pt-N co-modified TiO_2_ nanotube electrodes with various deposition times are shown in Figure 8. Under light illumination, it can be seen that the Pt-N modified nanotube electrode for the OTC solution had a higher degradation efficiency than a single TiO_2_ nanotube electrode. The photodeposition time of 60 min showed the best photocatalytic degradation efficiency, with a degradation rate of 56.17% within 60 min. The fitted curve of photocatalytic OTC degradation is shown in Figure 8b, and it can be shown that each nanotube’s degradation process complies with the equation of primary reaction kinetics, with the fastest degradation rate of 13.7 × 10^−3^ min^−1^ occurring at the 60 min deposition time. This pattern matches the outcomes of the electrochemical test work. 

**Figure 9:**
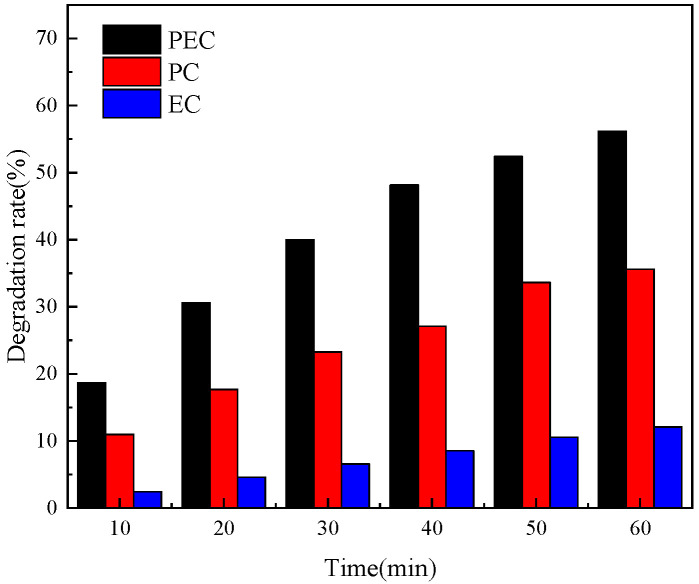
Comparison of the effect of PEC/EC/PC catalytic conditions for the degradation of OTC.

The rate tests for photocatalysis (PC), electrocatalysis (EC), and photoelectrocatalysis (PEC) were carried out on the nanotube electrode photodeposited for 60 min, in order to understand the impact of various catalytic circumstances on the catalytic activity of the Pt-N co-modified TiO_2_ nanotube electrode. The PC, EC, and PEC comparison curves for the nanotube electrode at a photodeposition time of 60 min are shown in Figure 9. The degradation rate of this nanotube electrode under three more catalytic conditions is shown to be in the following order: PEC > PC > EC. The degradation rates were 56.17% for PEC, 35.62% for PC, and 12.06% for EC, while the OTC degradation rate was increased by 20% when photoelectrocatalysis was performed compared with photocatalysis alone. It indicates that the photocatalytic activity of Pt-N-TiO_2_ nanotubes can be significantly improved by the auxiliary effect of the applied voltage. This result indicates that the applied voltage can promote the movement of photogenerated electrons generated at the anode of Pt-N-TiO_2_ nanotubes toward the cathode during photocatalysis, which improves the separation efficiency of electrons and holes and allows more active groups to participate in the reaction of oxidative degradation of OTC. The efficiency of OTC degradation under various catalytic circumstances can demonstrate the advantages of PEC.

**Figure 10:**
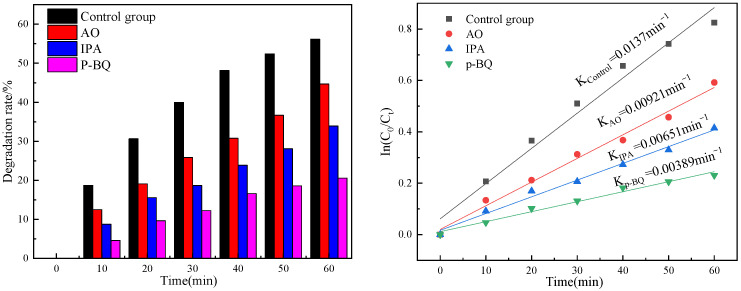
Effect of different trapping agents on photocatalytic OTC at Pt-N co-modified TiO_2_ nanotube electrodes and its reaction kinetics.

In the PEC system, the electrons in the valence band of the nanotube electrode absorb light energy and then leap from the valence band to the conduction band to generate reactive free radicals, which are essential oxide species leading to the degradation of pollutants [33]. By adding oxalic acid, it was possible to identify the primary active ingredients in the photoelectrocatalysis degradation of OTC by Pt-N co-modified TiO_2_ nanotubes. Ammonium oxalate (AO), isopropanol (IPA) and p-benzoquinone (q-BQ) were used to capture h^+^, •O_2_^−^ and •OH. The results are shown in Figure 10; the addition of AO, IPA and q-BQ had an inhibitory effect on OTC, the degradation rate at 60 min decreased from 56.17 % to 44.67%, 33.93%, and 20.57%, respectively. The primary kinetic reaction constants also decreased by 4.49 × 10^−3^ min^−1^, 7.08 × 10^−3^ min^−1^, and 7.56 × 10^−3^ min^−1^. The greatest decrease in the degradation rate was observed for P-BQ, followed by IPA and finally AO. It indicates that P-BQ has a significant inhibitory effect on the photocatalytic degradation process, and P-BQ is able to capture •O_2_^−^ through electron transfer in order to achieve the capture of •O_2_^−^, which causes the possible reason for the benzene ring breakage in OTC •O_2_^−^ preferentially attacking the benzene ring, and therefore it is thought that it may affect the benzene ring present in OTC. The degradation rate decreased by 11.5% and 35.6% after the addition of AO as well as IPA, respectively. This indicates the generation of cavities during the reaction, followed by the further conversion of a large amount of h^+^ into reactive radicals to degrade the OTC, rather than direct degradation. Therefore, we suggest that the photoelectrocatalytically generated •O_2_^−^ and •OH are important active oxides for OTC degradation, respectively.

## 4. Conclusions

SEM, XRD, PL, and XPS results demonstrated that the Pt and N elements were loaded onto the TiO_2_ nanotube electrode. The Pt-N co-modified TiO_2_ nanotube electrode with varied deposition duration was prepared by the photodeposition method as well as the plasma method. After alteration, the electrode’s morphological properties were preserved. Pt was present as a metal singlet. As a result, the noble metal tends to aggregate more with increased deposition time, decreasing overall light absorption. The best effect is deposited at 60 min, and the Pt-N co-modified TiO_2_ nanotube electrode light absorption is greatly increased in the visible range. A suitable amount of noble metal is photodeposition; too little noble metal deposition does not significantly improve catalytic efficiency, but too more noble metal deposition may also cause the noble metal to quickly recombination photogenerated electron-hole pairs, which will impact the photoelectrocatalysis efficiency. 

In the experiment of photoelectrocatalysis degradation of OTC, the Pt-N-TiO_2_ nanotube electrode with a photodeposition time of 60 min had excellent charge separation efficiency as well as excellent photoelectrocatalysis activity, and the degradation rate of photoelectrocatalysis degradation of OTC was 56.17%. The removal rate of OTC was in accordance with the primary kinetic law. The results of the trapping agent experiment are as follows: •O_2_^−^ and •OH are the main active oxide species leading to OTC degradation.

## Data Availability

All the data are available within the manuscript.
